# *PRRT2*-Related Epilepsy: From Self-Limited Infantile Epilepsy to Atypical Epilepsy Phenotypes

**DOI:** 10.1212/NXG.0000000000200267

**Published:** 2025-05-20

**Authors:** Madeline Komar, Jashanpreet Sidhu, Jiju Joseph, Ashna Kumar, David J.A. Callen, Ronit Mesterman, Rajesh Ramachandrannair, Kevin C. Jones, Brandon F. Meaney, Sonali Singh, Lyndsey McRae, Vann Chau, Suvasini Sharma, Elizabeth J. Donner, Rekha Aaron, Sumita Danda, Maya Thomas, Lokesh Saini, Gregory Costain, Sangeetha Yoganathan, Puneet Jain, Robyn Whitney

**Affiliations:** 1 Division of Neurology, Department of Paediatrics, McMaster University, Hamilton, Ontario, Canada;; 2 Division of Neurology, Department of Paediatrics, Hospital for Sick Children, University of Toronto, Ontario, Canada;; 3 Genetics and Genome Biology Program, SickKids Research Institute, Toronto, Ontario, Canada;; 4 Neurosciences and Mental Health Program, SickKids Research Institute, Toronto, Ontario, Canada;; 5 Pediatric Neurology Unit, Department of Neurological Sciences, Christian Medical College, Vellore, Tamil Nadu, India;; 6 Division of Neurology, Department of Paediatrics, AIIMS, Jodhpur, Rajasthan, India;; 7 Department of Medical Genetics, Christian Medical College, Vellore, Tamil Nadu, India;; 8 Division of Clinical and Metabolic Genetics, Hospital for Sick Children, University of Toronto, Ontario; and; 9 Department of Paediatrics, University of Toronto, Ontario, Canada.

## Abstract

**Background and Objectives:**

Pathogenic variants in the *PRRT2* gene cause self-limited infantile epilepsy (SeLIE). Recently, atypical epilepsy phenotypes have been described. We explore the phenotypic spectrum of *PRRT2*-related epilepsy through international collaboration.

**Methods:**

All children with epilepsy and either a pathogenic *PRRT2* variant or 16p11.2 microdeletion encompassing the *PRRT2* gene were included in this retrospective study. Details related to the epilepsy, comorbidities, genetic results, EEG/neuroimaging findings, and treatments are summarized.

**Results:**

Forty children were identified, and 24 were male (n = 24/40, 60%). The median age at seizure onset was 5 months (IQR 4, 6) (range 2–150 months). Bilateral tonic-clonic (BTC) (n = 22/40, 55%) and focal motor seizures with impaired awareness evolving to BTC seizures (n = 9/40, 23%) were most common. Thirty-six children (n = 36/40, 90%) had pathogenic *PRRT2* variants; 3 were homozygous, and 33 were heterozygous. Four children (n = 4/40, 10%) had 16p11.2 microdeletion. SeLIE was most common, diagnosed in 32 children with heterozygous *PRRT2* variants (n = 32/33, 97%), 3 children with homozygous *PRRT2* variants (n = 3/3, 100%), and 3 children with 16p11.2 microdeletion (n = 3/4, 75%). Atypical phenotypes were observed in 3 children with heterozygous *PRRT2* variants; 1 child evolved from SeLIE to infantile epileptic spasms, another developed spike-wave activation in sleep, and 1 developed focal epilepsy in adolescence. Medically refractory genetic generalized epilepsy and intellectual disability were diagnosed in 1 child with a whole-gene *PRRT2* deletion and 16p11.2 microdeletion. All children with homozygous *PRRT2* variants had SeLIE with movement disorders. Thirty-seven children (n = 37/40, 93%) were treated with antiseizure medications, and sodium channel blockers were effective in most (20/27 responded, 74%). The median age at seizure freedom was 9 months (IQR 3, 10) (range 3–168 months).

**Discussion:**

Pathogenic *PRRT2* variants are commonly associated with SeLIE. However, additional epilepsy phenotypes may be observed with heterozygous *PRRT2* variants. In individuals with heterozygous *PRRT2* variants, corresponding chromosomal microarray may be helpful to assess for concomitant 16p11.2 microdeletion, given the phenotypic overlap between the 2 conditions. Collection of additional cases is needed, however, to better understand the spectrum of epilepsy phenotypes associated with 16p11.2 microdeletion encompassing the *PRRT2* gene and homozygous and compound heterozygous *PRRT2* variants. Currently, precise genotype-phenotype relationships are lacking.

## Introduction

Our knowledge of genetically determined epilepsy has rapidly expanded over the past decade. There are now over 900 monogenic etiologies observed to be associated with epilepsy.^1^ Genetic forms of epilepsy are common in the neonatal and infantile period, especially in the case of developmental and epileptic encephalopathies (DEEs).^1,2^ In a recent multicenter study on the yield of rapid genome sequencing in neonatal/infantile seizures, 74% of infants with neonatal-onset seizures had an underlying genetic diagnosis, compared with 36% of infants with infantile-onset seizures, highlighting the vital contribution of genetics to epilepsy etiology in these age groups.^3^ Genetic discovery is crucial because it may allow for more tailored treatment approaches, provide information related to the underlying prognosis, and allow for relevant genetic counseling to occur.^2,3^

Pathogenic loss of function variants in the *PRRT2* gene are an important cause of self-limited infantile epilepsy (SeLIE) and other neurologic disorders such as hemiplegic migraine and *PRRT2*-associated paroxysmal movement disorders.^4,5^ Eighty percent of cases of SeLIE are secondary to pathogenic variants in the *PRRT2* gene, but less commonly, it may be secondary to pathogenic variants in *SCN8A* and *SCN2A*, for example.^4,5^ The *PRRT2* gene is located on chromosome 16 and encodes for proline-rich transmembrane protein 2, which has a role in exocytosis and neurotransmitter release.^4^ SeLIE often occurs among family members and is inherited in an autosomal dominant manner. However, de novo cases are also reported.^4-6^ The typical onset of seizures in SeLIE is around 6 months of age (range 2–8 months), and infants typically present with clusters of focal to bilateral tonic-clonic (BTC) seizures.^4,5^ The seizures usually abate by the age of 2 years and rarely may recur in adulthood.^4,6,7^ Furthermore, the seizures generally respond well to sodium channel blocker antiseizure medications (ASMs).^8^

More recently, there are single reports of pathogenic *PRRT2* variants causing additional epilepsy phenotypes, such as developmental and epileptic encephalopathy with spike-wave activation in sleep (DEE/EE-SWAS) and epilepsy of infancy with migrating focal seizures.^9-12^ It is unclear why some patients with pathogenic *PRRT2* variants present with a self-limited form of epilepsy while others develop a more severe epilepsy course. However, some of the phenotypic variability observed could be secondary to the effects of other genes within the 16p11.2 region where the *PRRT2* gene is located.^4,6^ Microdeletion within this region may lead to a broader range of clinical features and, therefore, complicate the phenotypic presentation of *PRRT2*-related disorders. In this study, we report an additional cohort of *PRRT2*-related epilepsy/seizures and highlight commonalities to previous descriptions as well as atypical features of the condition.

## Methods

### Standard Protocol Approvals, Registrations, and Patient Consents

Research ethics approval was obtained from the Hamilton Integrated Research Ethics Board (HIREB) at McMaster University in Hamilton, ON, Canada (HIREB# 16916). McMaster University acted as the coordinating site for each center, and ethics approval was obtained from each local research ethics board/institutional review board. Given its retrospective nature, the HIREB granted a waiver of informed consent for this study.

### Study Design, Inclusion Criteria, and Data Collection

All children (<18 years of age) with either a pathogenic/likely pathogenic *PRRT2* variant or 16p11.2 microdeletion encompassing the *PRRT2* gene and any history of epilepsy were included in this retrospective study. Children with homozygous *PRRT2* variants (i.e., both alleles carry identical variants) and biallelic *PRRT2* variants (i.e., both alleles are affected, but the exact configuration is uncertain; homozygous vs compound heterozygous) were also sought for inclusion. Children whose medical records were incomplete (i.e., genetic report not available) were excluded from the study, as well as those who had no history of epilepsy. Children with variants of uncertain significance (VUS) in *PRRT2* were also excluded. A chart review was conducted, and details were collected regarding the age at seizure onset, seizure types and frequency, International League Against Epilepsy epilepsy syndrome, neurologic comorbidities, EEG (EEG) and neuroimaging findings, genetic results, and treatments tried. Seizure onset was classified as infantile onset (i.e., before the age of 2 years), childhood onset (i.e., between 2 and 12 years), and adolescent onset (i.e., after 12 years of age). Genetic testing (e.g., next-generation sequencing gene panel test, exome sequencing, and genome sequencing) was performed on blood-derived DNA of patients at their respective sites. Chromosomal microarray was not performed in all cases. Sequence variants were classified using the American College of Medical Genetics criteria.^13^ All c. positions in the main text correspond to the canonical transcript (NM_145239.3; ENST00000358758.7) unless otherwise stated. The 16p11.2 copy number variations overlapped the 16p11.2 deletion syndrome region (OMIM: 611913) including the entirety of the *PRRT2* gene.

The coordinating site reviewed all data to ensure accuracy and completeness of the data set. Data were summarized using descriptive statistics, including median, range, and interquartile range for continuous variables and percentages and counts for categorical variables.

### Data Availability

Data used in this study will be made available on reasonable request for academic purposes, as guided by the local ethics committee.

## Results

### Demographics/Seizure History

**Figure 1:**
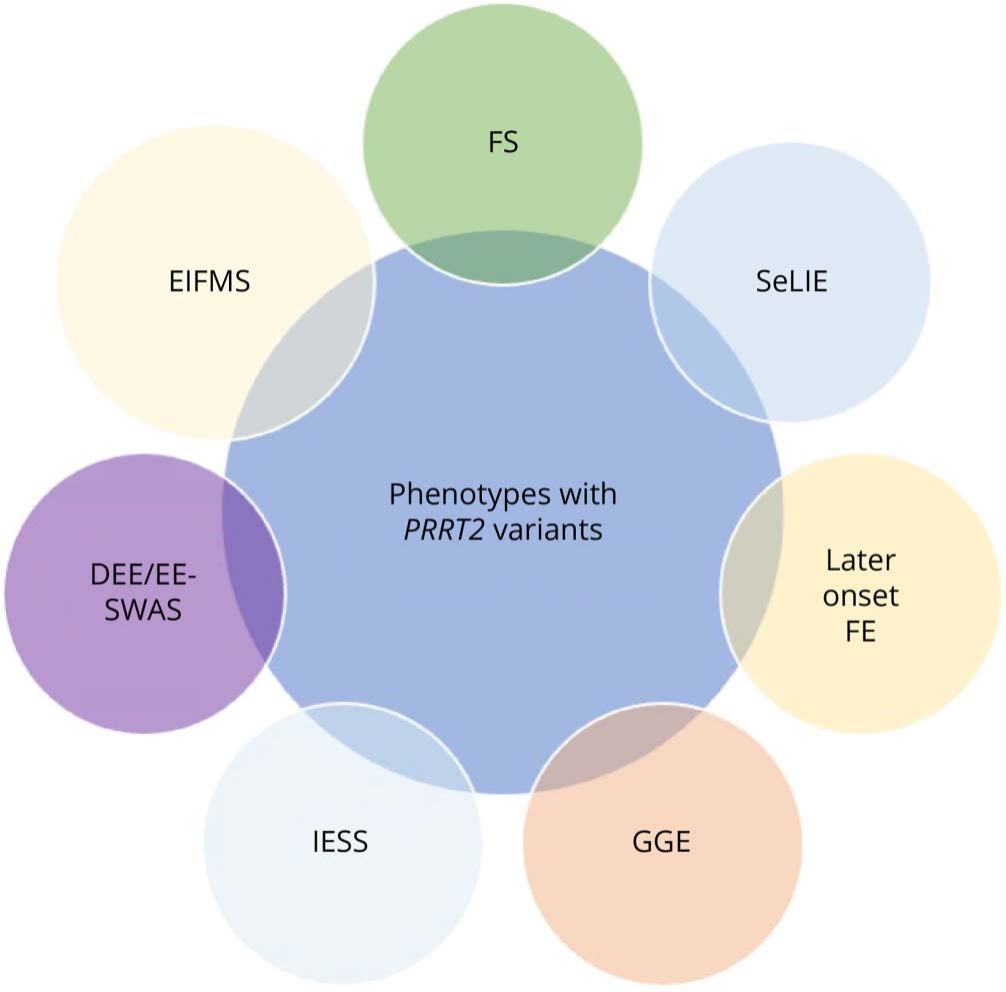
Spectrum of Epilepsy Phenotypes With *PRRT2 *Variants/16p11.2 Microdeletions Encompassing PRRT2 DEE/EE-SWAS = developmental and epileptic encephalopathy with spike-wave activation in sleep; EIFMS = epilepsy of infancy with migrating focal seizure; FE = focal epilepsy; FS = febrile seizure; GGE = genetic generalized epilepsy; IESS = infantile epileptic spasms syndrome; SeLIE = self-limited infantile epilepsy.

A total of 40 children were identified. Demographics and epilepsy characteristics are summarized in Table 1. Twenty-four children were male (n = 24/40, 60%). The median current age of the cohort was 3 years (IQR 3, 5.25) (range 0.8–16 years). The median age at seizure onset was 5 months (IQR 4, 6) (range 2–150 months). The most common seizure types were BTC seizures (n = 22/40, 55%), focal motor with impaired awareness evolving to BTC seizures (n = 9/40, 23%), generalized tonic seizures (n = 9/40, 23%), and focal motor seizures with impaired awareness (n = 5/40,13%). Seizure clustering was identified in 16 children (n = 16/40, 40%), and 3 children had a single seizure in their lifetime (n = 3/40, 8%). Seizure frequency was best categorized as monthly in 18 children (n = 18/40, 45%), infrequent (i.e., few seizures per year or single seizure) in 16 children (n = 16/40, 40%), weekly in 2 children (n = 2/40, 5%), daily in 2 children (n = 2/40, 5%), monthly then daily in 1 child (n = 1/40, 2.5%), and unknown in 1 child (n = 1/40, n = 2.5%). Eight children had a history of febrile seizures in addition to other seizure types (n = 8/40, 20%). SeLIE was the most common epilepsy syndrome in the cohort (n = 38/40, 95%). However, other epilepsy phenotypes were also observed, discussed herein. The spectrum of epilepsy phenotypes in our cohort and the literature is displayed in Figure 1. Neurologic comorbidities were present in 16 children (n = 16/40, 40%).

**Table 1:** Demographics and Epilepsy Characteristics of the Cohort

Case	Timing of Sz onset	Sz types	ILAE epilepsy syndrome	Movement disorder/migraine/other neurologic features	Genetics *PRRT2* NM_145239.2	ACMG/segregation	Current ASM	ASM most effective	Past ASM	Sz control: Sz free since age in mths	Follow-up duration
1	Infantile	FIAM to BTC BTC	SeLIE SWAS on EEG	None	c.649dupC (p.Arg217Profs*8)	Pathogenic, inherited	LTG	PHB and LTG both effective	PHB	Since 51 mths	56 mths
2	Infantile	BTC	SeLIE	Benign myoclonus of infancy	c.649dupC (p.Arg217Profs*8)	Pathogenic, inherited	None	PHB	PHB	Since 17 mths	32 mths
3	Infantile	FIAM to BTC	SeLIE	None	c.649dupC (p.Arg217Profs*8)	Pathogenic, inherited	None	PHB	PHB	Since 3 mths	18 mths
4	Infantile	FIAM	SeLIE	None	c.649dupC (p.Arg217Profs*8)	Pathogenic, inherited	None	PHB	PHB	Since 5 mths	12 mths
5	Infantile	FIANM	SeLIE	None	c.649dupC (p.Arg217Profs*8)	Pathogenic, inherited	LEV	PHB and LEV both effective	PHB	Since 5 mths	7 mths
6	Infantile	FIAM	SeLIE	None	c.649dupC (p.Arg217Profs*8)	Pathogenic, unknown	None	LEV	PHB, LEV	Since 41 mths	54 mths
7	Infantile	BTC	SeLIE	Migraines	c.649del (p.Arg217Glufs*12)	Pathogenic, unknown	None	NA	None	Since 6 mths	None lost to follow-up
8	Infantile	BTC	SeLIE	Migraines	c.649dupC (p.Arg217Profs*8)	Pathogenic, inherited	None	None	LEV, PHB	Since 19 mths	67 mths
9	Infantile	BTC	SeLIE	None	c.649dupC (p.Arg217Profs*8)	Pathogenic, inherited	None	NA	None	Since 11 mths	None
10	Infantile	BTC FIAM	SeLIE	None	c.649dupC (p.Arg217Profs*8)	Pathogenic, inherited	None	NA	None	Since 10 mths	6 mths
11	Childhood	BTC GAB	GGE	Intellectual disability Autistic features Obesity	16p11.2 GRCh38:chr16:g.(29614976_30215621)*; PPRT2*.(?_-21)_(*21_?)del, deletion of the entire coding gene PRRT2	Pathogenic, de novo	LTG CLB	None	None	Ongoing	4 mths
12	Infantile	FIAM to BTC ES	SeLIE → IESS	None	c.649dupC (p.Arg217Profs*8)	Pathogenic, inherited	RUF	RUF	PHB Prednisolone LEV, VGB	Since 11 mths	18 mths
13	Infantile	Focal Sz Febrile Sz	SeLIE	Dyskinesia	c.649dupC (p.Arg217Profs*8)	Pathogenic, inherited	OXC	OXC	LEV	Since 8 mths	20 mths
14	Infantile	FIAM to BTC GT FIANM	SeLIE	Central hypotonia	c.649dupC (p.Arg217Profs*8)	Pathogenic, inherited	OXC CLB	OXC	None	Since 9 mths	13 mths
15	Infantile	FIAM to BTC Febrile Sz	SeLIE	Central hypotonia	c.649del (p.Arg217Glufs*12)	Pathogenic, inherited	CBZ	CBZ	LEV PHB	Ongoing staring episodes	31.5 mths
16	Infantile	BTC Febrile Sz	SeLIE	Central hypotonia	16p11.2 GRCh38:chr16:g.(29600730_30188250)	Pathogenic, inherited	OXC	OXC	LEV	Since 9 mths	10 mths
17	Infantile	FIAM to BTC	SeLIE	None	c.649dupC (p.Arg217Profs*8)	Pathogenic, inherited	OXC	OXC	LEV	Since 5 mths	9 mths
18	Infantile	BTC	SeLIE	Dyskinesia Central hypotonia	c.511_512del (p.Leu171Valfs*2)	Likely pathogenic, inherited	OXC	OXC	LEV	Since 7 mths	7 mths
19	Infantile	BTC	SeLIE	Decreased tone	c.622delinsAA (p.Ser208Asnfs*17)	Pathogenic, inherited	OXC LEV	OXC and LEV both effective	None	Since 6 mths	5 mths
20	Infantile	BTC	SeLIE	None	c.649dupC (p.Arg217Profs*8)	Pathogenic, inherited	LEV	LEV	None	Since 12 mths	3 mths
21	Infantile	BTC Febrile Sz	SeLIE	None	c.649dupC (p.Arg217Profs*8)	Pathogenic, unknown	LAC CLB	LAC and CLB both effective	LEV	Since 10 mths	36 mths
22	Infantile	BTC GT	SeLIE	None	c.649dupC (p.Arg217Profs*8)	Pathogenic, inherited	LEV CLB LAC	LEV, CLB LAC, all effective	PHT	Since 6 mths	6 mths
23	Infantile	BTC	SeLIE	None	c.649dupC (p.Arg217Profs*8)	Pathogenic, unknown	CBZ	CBZ	PHT LEV	Since 12 mths	30 mths
24	Infantile	FIAM BTC Febrile Sz	SeLIE	None	c.649dupC (p.Arg217Profs*8)	Pathogenic, unknown	LEV CLB	LEV and CLB both effective	TPM	Since 8 mths	48 mths
25	Infantile	GT	SeLIE	None	c.649dupC (p.Arg217Profs*8)	Pathogenic, inherited	None	LEV and CLB both effective	PHT, LEV, CLB	Since 17 mths	60 mths
26	Infantile	FIAM to BTC Febrile Sz GT	SeLIE	Paroxysmal kinesigenic dyskinesia	c.649dupC (p.Arg217Profs*8) homozygous	Pathogenic, inherited	CBZ	VPA	VPA, PHT, PHB, CLB	Since 72 mths	180 mths
27	Infantile	GT BTC Febrile Sz	SeLIE	Autism spectrum disorder	c.971G > T (p.Gly324Val)	Pathogenic, unknown	LEV	LEV	PHT, CLB	Since 24 mths	60 mths
28	Infantile	FIAM to BTC BTC FIANM	SeLIE	None	c.223_224dup (p.Ala76ArgfsTer15)	Pathogenic, de novo	LEV	LEV	VPA, CLB	Since 9 mths	60 mths
29	Infantile	FIAM to BTC BTC	SeLIE	Choreoathetosis	c.971G > T (p.Gly324Val) homozygous	Pathogenic, unknown	CBZ, LEV, CLB, ACT	CBZ	PHB	Since 8 mths	30 mths
30	Infantile	GT	SeLIE	Dystonia	c.649dupC (p.Arg217Profs*8) homozygous	Pathogenic, inherited	CBZ	VPA	VPA	Since 12 mths	72 mths
31	Infantile	BTC	SeLIE	None	c.304G > T (p.Glu102Ter)	Pathogenic, de novo	None	VPA	LEV, VPA, OXC, CLB	Since 4 mths	36 mths
32	Infantile	GT	SeLIE	None	c.649dupC (p.Arg217Profs*8)	Pathogenic, unknown	CBZ	CBZ	LEV, CLB	Since 6 mths	36 mths
33	Infantile	BTC Febrile Sz	SeLIE	None	c.649dupC (p.Arg217Profs*8)	Pathogenic, inherited	LEV, CLB, ZNS	LEV, CLB, and ZNS effective	VPA, LEV, PHT	Since 12 mths	60 mths
34	Infantile	BTC	SeLIE	None	c.649dupC (p.Arg217Profs*8)	Pathogenic, unknown	LEV, OXC, CLB	OXC	VPA	Since 4 mths	24 mths
35	Infantile	GT	SeLIE	Dyskinesia	16p11.2 CRCh38:chr16:g.(?_29466576)_(30188687_?)del	Likely pathogenic, unknown	CBZ	CBZ	None	Since 10 mths	12 mths
36	Infantile	FIAM	SeLIE	None	c.649dupC (p.Arg217Profs*8)	Pathogenic, inherited	LEV, VPA	VPA	None	Since 7 mths	6 mths
37	Infantile	Clonic GA	SeLIE	None	16p11.2 GRCh38:Chr16:g.(?_29446802)_(30206900_?)del	Pathogenic, de novo	OXC	OXC	None	Since 8 mths	9 mths
38	Adolescence	FAM	None	Dyskinesia	c.604_607del (p.Ser202fs)	Pathogenic unknown	OXC	OXC	LEV	Since 168 mths	24 mths
39	Infantile	GT	SeLIE	None	c.649dupC (p.Arg217Profs*8)	Pathogenic, inherited	OXC	OXC	LEV	Since 7.5 mths	14.5 mths
40	Infantile	BTC	SeLIE	None	c.649dupC (p.Arg217Profs*8)	Pathogenic, de novo	OXC	OXC	None	Since 15 mths	19 mths

Abbreviations: ACMG = American College of Medical Genetics; ACT = acetazolamide; ASM = antiseizure medication; BTC = bilateral tonic-clonic; CAE = childhood absence epilepsy; CLB = clobazam; CBZ = carbamazepine; ES = epileptic spasm; FAM = focal aware motor onset; FIAM = focal impaired awareness motor onset; FIANM = focal impaired awareness nonmotor onset; FS = febrile seizure; GA = generalized atonic; GAB = generalized absence; GT = generalized tonic; ILAE = International League Against Epilepsy; LAC = lacosamide; LEV = levetiracetam; LTG = lamotrigine; mths = months; NA = not available; PHB = phenobarbital; PHT = phenytoin; OXC = oxcarbazepine; Ruf = rufinamide; TPM = topiramate; SeLIE = self-limited infantile epilepsy; SWAS = spike-wave activation in sleep; Sz = seizure; VGB = vigabatrin; VPA = valproate; ZNS = zonisamide.

### Genetics

**Figure 2:**
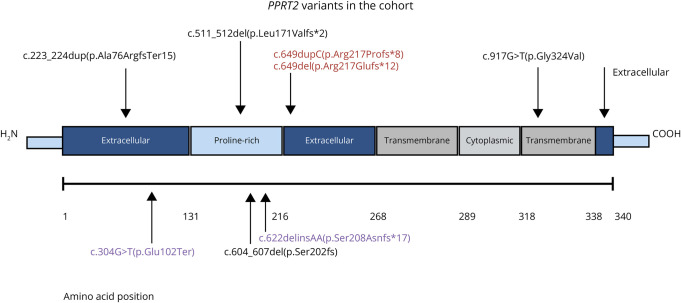
Distribution of the Genetic Variants in the Cohort Amino acids are displayed along the horizontal line (amino acids 1–340). The *PRRT2* gene encodes a protein with 340 amino acids. There is an extracellular domain, proline-rich domain, transmembrane, and cytoplasmic region. There is the N terminus and C terminus. The hotspot variants in *PRRT2* are highlighted in red; novel variants identified in this cohort are highlighted in purple. The remainder of the variants have been previously described in the literature. Created in BioRender. Whitney, R. (2024) BioRender.com/t74q858.

Nineteen children underwent exome sequencing (n = 19/40, 48%), 9 underwent genome sequencing (n = 9/40, 22%), 10 underwent epilepsy gene panel tests (n = 10/40, 25%), and 2 underwent targeted gene testing (n = 2/25, 5%). Thirty-six children (n = 36/40, 90%) had pathogenic/likely pathogenic *PRRT2* variants (3 were homozygous, 33 were heterozygous) (Table 1), and 4 children (n = 4/40, 10%) had 16p11.2 microdeletion encompassing the *PRRT2* gene (cases 11, 16, 35, and 37). A chromosomal microarray was completed in 1 case (1/40, 3%) (case 11), and the other cases of 16p11.2 microdeletion were diagnosed by genome/exome sequencing. The distribution of the genetic variants is shown in Figure 2. No other secondary genetic etiologies of epilepsy were identified in the cohort.

In 24 children, the *PRRT2* variant/16p11.2 microdeletion was inherited (n = 24/40, 60%); 5 children (n = 5/40, 13%) had de novo pathogenic *PRRT2* variants/16p11.2 microdeletions; and 11 had unknown inheritance patterns (n = 11/40, 27%). The c.649dup (p.Arg217Profs*8) was the most common *PRRT2* variant identified in 27 children (n = 27/36,75%). The recurrent c.649del (p.Arg217Glufs*12) was identified in 2 children (n = 2/36, 6%). Novel *PRRT2* variants were identified in 2 children, c.622delinsAA (p.Ser208Asnfs*17) and c.304G > T (p.Glu102Ter) (cases 19 and 31, respectively). The median age at genetic diagnosis was 8 months (IQR 6, 30) (range 1 month–170 months).

### Heterozygous *PRRT2* Variant Phenotype

Heterozygous *PRRT2* variants were found in 33 children in the cohort (n = 33/40, 83%). SeLIE was diagnosed in 32 children (n = 32/33, 97%). All children with heterozygous *PRRT2* variants were seizure free at the time of data collection with the exception of 1 child who had ongoing staring events of unclear significance (case 15). Two children with heterozygous *PRRT2* variants had seizures that persisted after 36 months of age (cases 1 and 6). Three atypical epilepsy phenotypes were observed associated with heterozygous *PRRT2* variants. One child with a heterozygous pathogenic *PRRT2* variant evolved from SeLIE (seizure onset before 6 months) to infantile epileptic spasms syndrome (IESS) at 6–7 months of age (case 12). This child had no significant neurologic comorbidities, and neuroimaging was noncontributory. At present time, the child is seizure free on rufinamide (RUF) (Table 1). Another child with a heterozygous pathogenic *PRRT2* variant who had SeLIE (seizure onset before 6 months) (case 1) developed SWAS beginning at 2–3 years of age, which was detected on routine EEG before medication taper. This child had no developmental stagnation/regression or neurologic comorbidities and was monitored over time clinically but was not treated as EE-SWAS, given the lack of neurodevelopmental regression. MRI of the brain revealed a right frontal heterotopia of uncertain significance. Currently, the child remains seizure free on lamotrigine (LTG) (Table 1). Focal-onset epilepsy in adolescence was diagnosed in another child with a heterozygous pathogenic *PRRT2* variant and dyskinesias (case 38). This child had no history of SeLIE, and EEG and neuroimaging studies were normal. Currently, the child remains seizure free on oxcarbazepine (OXC) at last follow-up (Table 1).

Neurologic comorbidities were present in 10 children with heterozygous *PRRT2* variants (10/33, 30%), and movement disorders (n = 4/10, 40%) (cases 2, 13, 18, and 38) and tone abnormalities (n = 4/10, 40%) (cases 14, 15,18, and 19) were the most common comorbidities (Table 1). One child was diagnosed with autism spectrum disorder (case 27).

### Homozygous *PRRT2* Variant Phenotype

Three children (cases 26, 29, and 30) had confirmed homozygous *PRRT2* pathogenic variants. All 3 children with homozygous *PRRT2* variants had a SeLIE phenotype with normal development and comorbid movement disorders (i.e., dyskinesias and dystonia). One child continued to have seizures until the age of 72 months (case 26) while the seizures in others stopped in infancy (case 29 and case 30).

### 16p11.2 Microdeletion Phenotype

Four children in the cohort (n = 4/40, 10%) had 16p11.2 microdeletion encompassing the *PRRT2* gene (cases 11, 16, 35, and 37) (Table 1). Three children with 16p11.2 microdeletion had a SeLIE phenotype with well-controlled seizures (cases 16, 35, and 37). However, 1 individual with a whole-gene *PRRT2* deletion and 16p11.2 microdeletion had childhood-onset medically refractory genetic generalized epilepsy (GGE) (case 11). This child was also diagnosed with intellectual disability with autistic features and obesity (case 11). Other comorbidities observed in children with 16p11.2 microdeletion included dyskinesias (case 35) and hypotonia (case 16). One child had no comorbidities (case 37).

### EEG and Neuroimaging Findings

EEG was completed in 37 children (n = 37/40, 93%), and abnormalities were found in 11 children (n = 11/37, 30%); the remainder (n = 26/37, 70%) were normal (Table 2). EEG was abnormal in 1 child with 16p11.2 microdeletion syndrome (case 11) and 10 children with heterozygous *PRRT2* variants (cases 1, 6, 8, 12, 13, 14, 15, 17, 19, and 24). All children with homozygous *PRRT2* variants had normal EEGs. Posterior predominant epileptiform discharges were most common and were found in 8 children with EEG abnormalities (n = 8/11, 73%) (cases 8, 12, 13, 14, 15, 17, 19, and 23), as summarized in Table 2. Neuroimaging was completed in 36 children (n = 36/40, 90%), and there were abnormalities observed in 11 children (n = 11/36, 31%) (cases 1, 12, 16, 17, 24, 27, 28, 33, and 35 (heterozygous *PRRT2*); cases 26 and 29 (homozygous *PRRT2*), as summarized in Table 2.

**Table 2:** EEG and Neuroimaging Findings of Cases With Confirmed Pathogenic *PRRT2* Variants

Case	EEG	Neuroimaging
1	Normal EEG in infancy, follow-up EEG in early childhood showed GSW, frequent right centrotemporal parietal SW with SWI between 50% and 70%	Normal MRI infancy; MRI in childhood: right frontal heterotopia
2	Normal EEGs in infancy	Normal MRI
3	None	None
4	None	None
5	Normal EEGs in infancy	Normal CT
6	Abnormal EEGs in infancy: SW right frontal central, right temporal SW, left temporal SW, 2 focal Szs from left frontal temporal region	Normal MRI
7	None	None
8	Abnormal EEG in infancy: SW left occipital and posterior temporal head region	MRI: left frontal extra-axial collection
9	Normal EEG in infancy	None
10	Normal EEG in infancy	Normal head ultrasound
11	Abnormal EEG in childhood: GSW, GPSW, generalized absence Sz with 3-Hz spike and wave	Normal MRI
12	Abnormal EEGs in infancy: right posterior IEDs; hypsarrhythmia; bilateral occipital IEDs; MISF	MRI in infancy: slight acceleration of myelination in the left occipital lobe with questionable obscuration of gray white matter differentiation; vigabatrin-induced changes, no structural abnormality
13	Abnormal EEGs in infancy: bilateral posterior IEDs, focal slowing bilateral temporal	Normal MRI
14	Abnormal EEG in infancy: bilateral posterior IEDs	Normal MRI
15	Abnormal EEGs in infancy: IEDs left posterior; focal slowing left posterior	Normal MRI
16	Normal EEG in infancy	MRI: slightly prominent extra-axial spaces
17	Abnormal EEG in infancy: frequent IEDs over left posterior quadrant	MRI: slightly prominent extra-axial spaces
18	Normal EEG in infancy	Normal MRI
19	Abnormal EEGs in infancy: Sz from left posterior, bilateral posterior IEDs; slowing right posterior, IEDs right posterior; bilateral posterior IEDs	Normal MRI
20	Normal EEG in infancy	Normal MRI
21	Normal EEG in infancy	Normal MRI
22	Normal EEGs in infancy	Normal MRI
23	Normal EEG in infancy	Normal MRI
24	Abnormal EEG in infancy: bilateral posterior IEDs	MRI: mild asymmetry lateral ventricles, mild high signal in white matter
25	Normal EEGs in infancy	Normal MRI
26	Normal EEGs in childhood	MRI: bilateral cerebellar atrophy
27	Normal EEGs in infancy	MRI: dysmorphic corpus callosum
28	Normal EEGs in infancy/childhood	MRI: bilateral cerebellar atrophy
29	Normal EEGs in infancy/childhood	MRI: widening of Sylvian fissure bilateral, left insula appeared less sulcated
30	Normal EEGs in infancy/childhood	Normal MRI
31	Normal EEGs in infancy	Normal MRI
32	Normal EEGs in infancy	Normal MRI
33	Normal EEGs in infancy	MRI: bilateral white matter loss, mild corpus callosum thinning
34	Normal EEGs in infancy	Normal MRI
35	Normal EEGs in childhood	MRI: bilateral globus pallidus and parietal occipital T2 hyperintensities
36	Normal EEGs in infancy	Normal MRI
37	Normal EEGs in infancy	Normal MRI
38	Normal EEGs in adolescence	Normal MRI
39	Normal EEGs in infancy	Normal MRI
40	Normal EEGs in infancy	Normal MRI

Abbreviations: GPSW = generalized polyspike and slow wave; GSW = generalized spike wave; IEDs = interictal epileptiform discharges; MISF = multiple independent spike foci; mths = months; SW = sharp wave; SWI = spike-wave index; Sz = seizure; yrs = years.

### ASMs

Thirty-seven children (n = 37/40, 93%) were treated with ASMs (Table 1). The median number of ASMs tried in the entire cohort was 2 (IQR 1.75, 3). A variety of ASMs were used. The most common ASMs tried were as follows: sodium channel blockers (n = 27/37, 73%), levetiracetam (LEV) (n = 27/37, 73%), clobazam (CLB) (n = 13/37, 35%), phenobarbital (PHB) (n = 11/37, 30%), and valproate (VPA) (n = 8/37, 22%). Sodium channel blockers were most effective in 20 children who had been prescribed them (n = 20/27, 74%). LEV was most effective in 10 children (n = 10/27, 37%), PHB in 5 children (n = 5/11, 45%), VPA in 4 children (n = 4/8 50%), and CLB in 5 children (5/13, 38%)

Thirty-eight children (n = 38/40, 95%) were seizure free at the time of data collection, and 1 child continued to have ongoing seizures (case 11, 16p11.2 microdeletion); another child had staring events, although it was unclear whether these were epileptic (case 15, heterozygous *PRRT2*). The median age at which seizure freedom was obtained was 9 months (IQR 3, 10) and ranged from 3 months to 168 months.

## Discussion

We describe additional 40 cases of epilepsy associated with pathogenic *PRRT2* variants/16p11.2 microdeletions and supplement what is known about this condition. Similar to previous cohorts, the most common epilepsy phenotype was SeLIE with clusters of focal motor seizures with or without evolution to BTC seizures and BTC seizures alone.^4,6,8,9,12^ Seizure clustering was observed in half of the cohort and occurred at similar rates in the literature.^4,8,12^ EEG was normal in two-thirds, and focal epileptiform discharges over the posterior head regions were most common. Posterior predominant epileptiform discharges have recently been described in the literature with pathogenic *PRRT2* variants.^14^ Heterozygous *PRRT2* variants in our cohort were most commonly associated with SeLIE with seizure resolution before 36 months of age. However, atypical epilepsy phenotypes were also observed, including evolution from SeLIE to IESS, adolescent-onset focal epilepsy (FE), and SeLIE with evolution to SWAS on EEG. Two children with heterozygous *PRRT2* variants and SeLIE also continued to have persistent seizures after 36 months of age. 16p11.2 microdeletion encompassing the *PRRT2* gene was associated with a variable epilepsy/neurologic phenotype in our cohort ranging from SeLIE with well-controlled seizures to childhood-onset medically refractory GGE with intellectual disability and autistic features. Homozygous *PRRT2* variants in our cohort were associated with SeLIE with normal development and comorbid movement disorders. In addition, 2 novel variants in *PRRT2* were observed in our cohort.

IESS is rarely associated with heterozygous variants in the *PRRT2* gene. Two cases are described in the literature: one of an infant with a variant of uncertain significance (VUS) in the *PRRT2* gene and IESS and another infant with a pathogenic *PRRT2* variant who evolved from SeLIE at 4 months of age to IESS at 7 months, similar to our case (case 12).^9,15^ Evolution to EE-SWAS is described in 2 cases in the literature; each case evolved from SeLIE to EE-SWAS at 5 years of age with prominent centrotemporal epileptiform discharges and developmental stagnation on neuropsychology assessment.^10^ By contrast, our case presented with early-onset SWAS on EEG at the age of 2–3 years with both generalized and focal interictal epileptiform discharges (right central parietal) but had no slowing or regression in development and did not meet diagnostic criteria for EE-SWAS. Neuroimaging was initially normal in infancy, but in mid-childhood, it showed a small heterotopia adjacent to the caudate in the right frontal lobe. It is possible that this neuroimaging finding may have contributed to the focal EEG findings along with the *PRRT2* variant or that it was incidental. The mechanism surrounding the evolution from SeLIE to EE-SWAS with pathogenic *PRRT2* variants is unknown. However, it has been previously hypothesized that the wide cortical and subcortical expression of *PRRT2* could result in focal EEG patterns and evolution to more diffuse EEG patterns seen in EE-SWAS. We propose that this mechanism may also influence the development of IESS.^10^

In addition, 1 child with a heterozygous *PRRT2* variant in our cohort (case 38) had adolescent-onset FE with no history of SeLIE. This child also had a history of dyskinesias. Neuroimaging and EEG were both normal, and the seizures responded to OXC. Overall, there are few reports of adult-onset generalized epilepsy with pathogenic *PRRT2* variants in the literature. Moreover, recurrent seizures have rarely been described in adulthood after an atypical SeLIE course.^7,16,17^ However, adolescent/adult-onset FE without a history of SeLIE is not well described. An adult female patient with focal-onset temporal lobe epilepsy and paroxysmal kinesigenic dyskinesias/dystonia (PKD) who carried the same pathogenic *PRRT2* variant as our case, c.604_607del (p.Ser202fs), was recently reported.^18^ This patient also had no history of SeLIE and responded to LTG.^18^ However, it is unclear whether the rare frameshift variant found in both patients predisposed to development of later-onset FE. Nevertheless, our additional case illustrates the spectrum of epilepsy that may occur with pathogenic *PRRT2* variants, including later-onset FE.

Microdeletions in 16p11.2 encompassing the *PRRT2* gene are often associated with a SeLIE phenotype.^19^ An atypical course of SeLIE has been described in some infants with 16p11.2 microdeletions with seizure recurrence after remission, earlier seizure onset, and later age at seizure remission.^19^ However, in most cases, the seizures resolve in infancy.^19^ Three of 4 cases with 16p11.2 microdeletions had SeLIE with no atypical features. However, 1 child (case 11) presented with medically refractory tonic-clonic and absence seizures in late childhood and had no history of SeLIE. This child was subsequently diagnosed with a GGE with concomitant intellectual disability and autistic features. Intellectual disability and autism spectrum disorder are both associated with 16p11.2 microdeletion syndrome.^19^ There are isolated reports of 16p11.2 microdeletions being related to other epilepsy phenotypes, such as epilepsy with myoclonic absences.^19^ However, given the small case numbers and the short period of follow-up in our study, we were not able to assess for evolution to other epilepsy phenotypes in this group and it is also possible that other neurologic comorbidities were underestimated. Microdeletions in 15q11.2 and 16p11.3 have been more frequently identified in individuals with GGE. However, copy number variants in 16p11.2 are less reported, and thus, the spectrum of the epilepsy is less well known.^19-21^

Given the known association between 16p11.2 microdeletion and a more severe neurologic presentation (i.e., intellectual disability and autism) as demonstrated in our case (case 11), it is important that individuals with 16p11.2 microdeletion undergo routine neurodevelopmental surveillance and be followed for the development new seizures/epilepsy types.^19^ In addition, owing to the phenotypic overlap between 16p11.2 microdeletion and *PRRT2*-related disorders, it may be helpful to pair *PRRT2* genetic testing with microarray analysis to look for 16p11.2 microdeletion syndrome, given the broader clinical phenotype that can be associated with it. In our case (case 11), paired gene panel with microarray led to the diagnosis of 16p11.2 microdeletion syndrome. There are few cases (total = 4) in the literature that report individuals with both heterozygous *PRRT2* variants and 16p11.2 microdeletion; all had epilepsy, and learning disabilities, intellectual disability, and autism spectrum disorder were seen in half of the cases.^22-24^

There are few reports of patients with homozygous and compound heterozygous *PRRT2* variants in the literature.^22,25^ These cases have been reported to have a more severe epilepsy phenotype than SeLIE (i.e., daily seizures despite ASM treatment and seizures occurring later in childhood/adulthood), intellectual disability, behavioral difficulties, abnormal neuroimaging, and more prolonged movement disorders (i.e., PKD).^22,25^ Homozygous and compound heterozygous deleterious *PRRT2* variants have also been found to be associated with prolonged episodes of episodic ataxia and cerebellar atrophy on neuroimaging.^22,25^ However, none of our cases with homozygous *PRRT2* variants had intellectual disability, behavioral difficulties, or ataxia (cases 26, 29, and 30). All 3 cases had movement disorders including dystonia, choreoathetosis, and PKD. Two children had SeLIE with seizure remission in early infancy while another child continued to have seizures until mid-childhood (case 26). Absence seizures were not observed in our cases with homozygous *PRRT2* variants but have been rarely described in the literature.^25^ Mild cerebellar atrophy was observed in one of our cases (case 26), and EEGs were normal. Three siblings from Sudan with homozygous *PRRT2* variants also presented SeLIE and had no additional neurologic features.^26^ However, given the small number of cases in our series and the literature, additional studies are needed to better understand the epilepsy phenotype associated with homozygous and compound heterozygous *PRRT2* variants.

Movement disorders were the most common neurologic comorbidity and occurred in one-fifth of the cohort. However, it is possible that the number of children with movement disorders was underestimated, given the lack of longitudinal follow-up in our cohort. Dyskinesias, dystonia, and choreoathetosis were observed. One child (case 2) with SeLIE and a heterozygous *PRRT2* variant had nonepileptic head drops consistent with benign myoclonus of infancy, which has been previously described in only 1 case in the literature.^27^ Similar to the literature, cognitive development was normal in all children,^17^ except for 1 child with 16p11.2 microdeletion (case 11) who had intellectual disability with autistic features. Furthermore, 1 child with a heterozygous *PRRT2* variant and SeLIE (case 27) was diagnosed with autism spectrum disorder. This child was diagnosed through exome sequencing, however, and chromosomal microarray was not performed to assess for 16p11.2 microdeletion. Of the children with 16p11.2 microdeletion, 1 child had obesity, which is a known comorbidity, and another child had hypotonia, which has also been previously reported in the literature (case 16).^28^ The extent of comorbidities in 16p11.2 microdeletion in our cohort is likely underestimated, given that 2 cases (cases 16 and 37) were still infants.

Overall, several different ASMs were used in our cohort. LEV, PHB, CLB, VPA, and sodium channel blockers were most commonly prescribed (i.e., carbamazepine, OXC, phenytoin, and RUF). ASMs other than sodium channel blockers were likely used because the genetic diagnosis typically lagged behind clinical seizure onset (i.e., median 8 months for genetic diagnosis vs 5 months for seizure onset).^8^ Sodium channel blockers were effective in most children prescribed them, which has also been described in the literature.^8,17^ Previous studies have shown that individuals treated with LEV and PHB required additional ASMs because of inadequate seizure control. However, some children in our cohort responded to these agents.^8^ It is difficult to know whether the response to other ASMs, such as PHB, was secondary to the effect of the ASM alone or was related to the self-limiting nature of epilepsy in children with SeLIE. Given the small number, we were not able to assess difference in response to ASMs based on the underlying genotype.

Finally, we could not identify any precise genotype-phenotype relationships in our cohort, nor are they well established in the literature.^29^ Most children in our cohort had the recurrent hotspot *PRRT2* variant, c.649dupC (p.Arg217Profs*8). The other hotspot variant, c.649delC (p.Arg217Glufs*12), was less common in our cohort.^4^ Most frameshift variants in *PRRT2* result in a truncated protein that lacks both the C-terminal and transmembrane domain.^17,30^ Truncating variants occur throughout the gene while missense variants impair plasma membrane localization and cluster around the C-terminus.^17,30^ It has been proposed that whole-gene *PRRT2* deletions/haploinsufficiency may be associated with a more severe phenotype. This finding is supported by our single case with a whole-gene *PRRT2* deletion (case 11) who was diagnosed with medically refractory GGE.^12^ Homozygous/compound heterozygous *PRRT2* variants also disrupt the PRRT2 protein more than heterozygous variants and have been reported to cause a more severe phenotype.^17,22,25^ However, the 3 children with homozygous *PRRT2* variants in our cohort did not display a severe epilepsy/neurologic phenotype.^22,25^ However, overall, our small sample size limited our ability to make meaningful genotype-phenotype correlations. Furthermore, genotype-phenotype relationships are not precise with pathogenic *PRRT2* variants, and it is unclear why some patients present with a self-limited epilepsy course and others develop DEEs.^29^ For example, the child who developed IESS shared the same genotype c.649dupC (p.Arg217Profs*8) as several children in the cohort who were diagnosed with SeLIE alone. Likely, other unknown genetic mechanisms (modifier genes, epigenetics) or environmental influences could modify the epilepsy phenotype.^17,29,31^ Moreover, variants in the *PRRT2* gene display pleiotropy, a high degree of variable expressivity, and incomplete penetrance (50%–90%).^17,28^ Understanding the spectrum of epilepsy in patients with *PRRT2* variants is important when counseling patients and caregivers.

Our study has limitations, including that it was a retrospective study and had a small sample size. We could not look for common or rare modifiers of gene expression. Seizure types, frequencies, and treatment responses to ASMs were based on parental reports, which may be more subjective. Although genetic testing did not identify a dual diagnosis in any of the children, it is possible that other unknown genetic mechanisms/conditions modified the phenotype. Given that most children in the cohort did not undergo additional chromosomal microarray, it is possible that the number of individuals with 16p11.2 microdeletion was underestimated. Moreover, nearly half of the cohort underwent exome sequencing and it is possible that some small microdeletions in 16p11.2 may have been missed because of the limitations in detecting copy number variants with exome sequencing. The extent of neurologic comorbidities is likely underestimated in our cohort, given that some children were still infants. We also cannot rule out subtle structural changes that may have been missed during initial neuroimaging and could have contributed to the epilepsy phenotype. We were unable to obtain specific details on the epilepsy phenotype in relatives. Long-term follow-up was limited, and we were not able to assess the natural history of the epilepsy over time for all patients in our cohort.

In summary, we demonstrate that although pathogenic *PRRT2* variants are most commonly associated with a SeLIE phenotype, atypical features may occur with seizures persisting later into childhood or the development of additional epilepsy phenotypes in others, such as IESS. Collection of additional cases, however, is needed to better understand the epilepsy phenotypes associated with 16p11.2 microdeletions encompassing the *PRRT2* gene and homozygous and compound heterozygous *PRRT2* variants. In individuals with heterozygous *PRRT2* variants, corresponding chromosomal microarray may be helpful to assess for concomitant 16p11.2 microdeletion, given the phenotypic overlap between the 2 conditions. Ongoing surveillance of individuals with 16p11.2 microdeletion syndrome is important to screen for additional neurologic comorbidities and epilepsy phenotypes over time. Overall, sodium channel blockers remain an effective treatment for epilepsy related to pathogenic *PRRT2* variants. Currently, precise genotype-phenotype relationships are lacking.

## Glossary

ASM: antiseizure medication
BTC: bilateral tonic-clonic
CLB: clobazam
DEEs: developmental and epileptic encephalopathies
DEE/EE-SWAS: developmental and epileptic encephalopathy with spike-wave activation in sleep
FE: focal epilepsy
HIREB: Hamilton Integrated Research Ethics Board
IESS: infantile epileptic spasms syndrome
LEV: levetiracetam
LTG: lamotrigine
OXC: oxcarbazepine
PHB: phenobarbital
PKD: paroxysmal kinesigenic dyskinesias/dystonia
RUF: rufinamide
SeLIE: self-limited infantile epilepsy
VPA: valproate
